# Aβ Seeding as a Tool to Study Cerebral Amyloidosis and Associated Pathology

**DOI:** 10.3389/fnmol.2019.00233

**Published:** 2019-10-02

**Authors:** Marina Friesen, Melanie Meyer-Luehmann

**Affiliations:** ^1^Department of Neurology/Neurodegeneration, Medical Center—University of Freiburg, Freiburg, Germany; ^2^Faculty of Medicine, University of Freiburg, Freiburg, Germany; ^3^Faculty of Biology, University of Freiburg, Freiburg, Germany; ^4^Center for Basics in NeuroModulation (NeuroModulBasics), Faculty of Medicine, University of Freiburg, Freiburg, Germany

**Keywords:** Alzheimer’s disease, cerebral amyloidosis, amyloid plaques, Aβ seeding, cross-seeding

## Abstract

Misfolded proteins can form aggregates and induce a self-perpetuating process leading to the amplification and spreading of pathological protein assemblies. These misfolded protein assemblies act as seeds of aggregation. In an *in vivo* exogenous seeding model, both the features of seeds and the position at which seeding originates are precisely defined. Ample evidence from studies on intracerebal injection of amyloid-beta (Aβ)-rich brain extracts suggests that Aβ aggregation can be initiated by prion-like seeding. In this mini-review article, we will summarize the past and current literature on Aβ seeding in mouse models of AD and discuss its implementation as a tool to study cerebral amyloidosis and associated pathology.

## History of Seeding: From *in vitro* to *in vivo*

Protein aggregation is a common feature of many neurodegenerative diseases that is assumed to play a central role in the pathogenesis. The aggregation of Aβ has been described as a nucleation-dependent polymerization process, including an initial slow nucleation phase, also called lag-phase, followed by a rapid growth phase (Jarrett and Lansbury, 1993; Jarrett et al., 1993; Harper and Lansbury, 1997; Walsh et al., 1997). The nucleus or seed formation in the nucleation phase is the rate-limiting step and thermodynamically unfavorable (Jarrett and Lansbury, 1993). The addition of stable seeds, e.g., aggregates generated by fragmentation of fibrils (Jarrett and Lansbury, 1993; Falsig et al., 2008; Knowles et al., 2009; Xue et al., 2009), accelerate the polymerization process and shorten significantly the lag-phase in a process termed “seeding” (Jarrett and Lansbury, 1992, 1993; Harper and Lansbury, 1997). These preformed seeds serve as a template for polymerization of respective aggregates (Jarrett and Lansbury, 1992) and can either have the same nature as the nuclei leading to a homologous seeding or be made from a different protein inducing heterologous seeding (Morales et al., 2009, 2013). Several *in vitro* and *in vivo* aggregation studies have provided the essential proof for a seeded-nucleation model of Aβ (Jarrett et al., 1993; Lomakin et al., 1996, 1997; Harper and Lansbury, 1997; Kane et al., 2000; Walker et al., 2002; Petkova et al., 2005; Meyer-Luehmann et al., 2006; Knowles et al., 2009; Paravastu et al., 2009; Eisele et al., 2010; Cohen et al., 2013).

The nucleation-dependent polymerization process gives rise to several Aβ assemblies such as oligomers, protofibrils and fibrils (Glenner and Wong, 1984; Harper et al., 1997; Lambert et al., 1998). Soluble Aβ oligomers were proposed to be the most toxic species of Aβ (Walsh et al., 2002; Wang et al., 2002; Cleary et al., 2005; Lesné et al., 2006; Townsend et al., 2006; Shankar et al., 2008), as it was already demonstrated for small assemblies of the prion protein (PrP; Silveira et al., 2005). Intracerebroventricular injections of media containing naturally secreted Aβ oligomers into rats led to inhibition of hippocampal long-term potentiation (LTP) and disrupted cognitive function (Walsh et al., 2002; Cleary et al., 2005). Moreover, soluble Aβ oligomers such as dimers isolated from human AD brains and Aβ*56 isolated from APP-transgenic (APP-tg) mouse brain were shown to impair synaptic plasticity and memory when administered to rodents and rat hippocampal slices (Lesné et al., 2006; Shankar et al., 2008). Hyperaggregation of soluble Aβ into higher aggregates was shown to be protective against Aβ mediated toxicity (Cohen et al., 2009). Despite the fact that soluble oligomers represent the most toxic species of Aβ, the presence of insoluble Aβ amyloid plaques leads to disruption of neocortical synaptic transmission, neuronal deformation and neuronal dysfunction (Stern et al., 2004; Tsai et al., 2004; Meyer-Luehmann et al., 2008, 2009).

The intracerebral injections of PrP-containing human brain homogenates into animals have led to the discovery of the transmissible prion disease approach (Gajdusek et al., 1966; Gibbs et al., 1968; Gajdusek, 1977; Hadlow et al., 1980; Prusiner, 1982). Since PrP and the Aβ peptide share similar biological and molecular features regarding the pathogenic self-assembly, misfolding and spreading within the brain, the question whether this concept could also be applied to other neurodegenerative diseases such as Alzheimer’s disease with Aβ peptide as potential trigger of the disease has been under discussion for quite some time (Prusiner, 1984; Rasmussen et al., 2017a).

Initial *in vivo* Aβ seeding experiments were performed by intracerebral inoculation of brain extract from AD patients in non-human primates that yielded inconsistent results (Goudsmit et al., 1980; Manuelidis and Manuelidis, 1991). Later attempts to seed Aβ pathology were successful in wild-type marmosets (Baker et al., 1993, 1994; Ridley et al., 2006), a New World monkey that express human-type sequence of Aβ (Heuer et al., 2012). Intracerebral infusion of brain tissue material from an AD patient resulted in Aβ deposits that could not be observed in control animals (Baker et al., 1993, 1994; Ridley et al., 2006). Interestingly, the distribution pattern of exogenously induced plaques was similar to those of elderly uninjected controls that developed cerebral amyloidosis (Maclean et al., 2000). However, the use of marmosets as a model for AD was considered impractical (Baker et al., 1993, 1994; Maclean et al., 2000; Ridley et al., 2006).

It is worth highlighting that compared to other mouse models for α-synuclein and tau, it is not possible to induce cerebral amyloid deposition in non-transgenic mice within its normal life span (Meyer-Luehmann et al., 2006; Luk et al., 2012; Guo et al., 2016), due to three amino acid difference between the mouse- and human-derived Aβ sequence (Otvos et al., 1993).

The strongest piece of evidence for “prion-like” seeding of misfolded Aβ aggregates *in vivo* was documented in experiments carrying out the inoculation of diluted brain extracts derived from confirmed AD patients into young, pre-depositing APP-tg mice (Kane et al., 2000; Walker et al., 2002; Meyer-Luehmann et al., 2006). APP-tg mice inoculated with AD brain extracts displayed remarkable Aβ deposition as well as mice injected with brain homogenate from control patients due to similar Aβ production levels (Kane et al., 2000; Walker et al., 2002; Meyer-Luehmann et al., 2006; Szaruga et al., 2015), while attempts to seed Aβ pathology *in vivo* using cerebrospinal fluid (CSF) from AD patients failed, although Aβ concentrations were significantly higher than the ones present typically in brain homogenates (Fritschi et al., 2014b). Whether some cofactors or a specific conformation of Aβ is missing in CSF compared to brain homogenates is currently not clear (Fritschi et al., 2014b). Further studies confirmed that APP-tg mouse brain extracts were equally efficient to seed Aβ aggregation in APP-tg mice as the ones prepared from human AD brains (Meyer-Luehmann et al., 2006; Eisele et al., 2009; Watts et al., 2011; Morales et al., 2012; Rosen et al., 2012). Most of those Aβ seeding studies are carried out in tg mice overexpressing mutant human APP, although Aβ deposits can also be induced *de novo* in rodents after comparable longer incubation periods that would never exhibit Aβ plaque pathology spontaneously within their normal lifespan (Morales et al., 2012; Rosen et al., 2012). This is a strong indication that exogenously applied seeds act as a template for misfolding of endogenous Aβ and that seeding is not solely promoting the premature deposition of amyloid. Importantly, the overexpression of APP seems not essential for the prion-like propagation of seeded Aβ aggregates within the brain (Ruiz-Riquelme et al., 2018).

## Nature of Aβ Seeds and Seed-Induced Aβ Deposits

The injection of autopsy-derived, brain extracts from young control individuals into APP-tg mice with no seed-induced Aβ deposits suggested that the presence of Aβ in the brain extract is crucial for *in vivo* seeding (Kane et al., 2000; Walker et al., 2002). To substantiate this hypothesis, depletion of Aβ and Aβ aggregates from the brain extracts prevented induction of Aβ deposits in host mice as well as formic acid treatment of the inoculum (Meyer-Luehmann et al., 2006; Duran-Aniotz et al., 2014). On the other hand, synthetic, multimeric Aβ fibrils have also been used to determine the essential seeding factor. Original experiments using synthetic Aβ in different composition displayed only poor seeding capacity (Meyer-Luehmann et al., 2006), while higher amounts of synthetic Aβ fibrils were indeed seed-competent but not as efficient as Aβ-containing human or mouse brain homogenates (Stöhr et al., 2012, 2014). Using hippocampal slice culture model, Novotny et al. revealed that synthetic Aβ can be converted into seeds able to induce β-amyloidosis *in vivo* (Novotny et al., 2016). Whether a particular conformation of the Aβ or additional factors is needed to induce seeding is not yet clear. To assess the stability of Aβ seeds *in vivo*, APP-null mice have been used as recipient mice that fail to induce Aβ pathology because of absent Aβ production. Six-months post-incubation, brain homogenates of these mice successfully induced Aβ deposition in APP-tg hosts, suggesting that Aβ seeds are highly robust and are able to retain their seeding activity for months (Ye et al., 2015a). Furthermore, Aβ-rich extracts prepared from human AD and APP-tg mice brains after formaldehyde treatment for 1–2 years were still able to seed Aβ deposits in APP-tg mice (Fritschi et al., 2014a). Nonetheless, the question still remains which Aβ species in brain homogenates is essential for the seeding activity in APP-tg mice and what cofactors are required. Indeed, another study revealed that Aβ seeds do not consist of only one type of Aβ aggregate, but are rather a mixture of small soluble or insoluble (in 100,000× *g* ultracentrifuged supernatant or pellet fraction) and proteinase-K (PK)-sensitive or PK-resistant Aβ species (Langer et al., 2011). Interestingly, sonication and thus fragmentation of the insoluble fraction into smaller and more soluble Aβ seeds, enhanced the seeding activity of the inoculum, consistent with results from fragmentation studies (Jarrett and Lansbury, 1993; Falsig et al., 2008; Knowles et al., 2009; Xue et al., 2009). In line with the previous mentioned studies, Aβ oligomers seem to be important for the initiation of Aβ aggregation especially for the early phase of the seeding process (Katzmarski et al., 2019). According to another study, the Aβ seeding potency of the brain extracts is highest at the very early stage of cerebral amyloidosis (Ye et al., 2017).

As demonstrated before, Aβ can aggregate into polymorphic shapes (Fändrich et al., 2009; Levine and Walker, 2010; Eisenberg and Jucker, 2012). Aβ isolated from AD patients were shown to trigger synthetic Aβ to adopt corresponding structural “strains” (Lu et al., 2013; Qiang et al., 2017). Also molecular differences in Aβ between non-demented and AD cases were pointed out (Piccini et al., 2005; Portelius et al., 2015), supporting the hypothesis of defined Aβ “strains” that probably correspond to the development of AD pathology. “Strain-like” variations of Aβ were observed in *in vitro* (Petkova et al., 2005; Nilsson et al., 2007; Paravastu et al., 2008; Meinhardt et al., 2009; Spirig et al., 2014) as well as *in vivo* studies using AD mouse models (Meyer-Luehmann et al., 2006; Heilbronner et al., 2013; Stöhr et al., 2014; Watts et al., 2014; Condello et al., 2018). Moreover, these “strain-like” variations in the molecular structure of Aβ aggregates were shown to be transmissible between APP-tg donor and recipient (Meyer-Luehmann et al., 2006; Heilbronner et al., 2013). Interestingly, the morphology and the biochemical composition of the seed-induced Aβ deposits represented histopathological features of the plaques present in donor and host mice. The respective brain region and thus the local environment seem to play a crucial role with regard to the morphology of the exogenously induced Aβ plaques (Eisele et al., 2009; Ye et al., 2015b). While infusion of brain homogenate into the hippocampus yielded compact and diffuse plaques, injections into striatum led to the formation of merely diffuse plaques (Eisele et al., 2009). Finally, “strain-like” features of Aβ aggregates present in different human familial AD cases could be partially recapitulated in mouse models by exogenous seeding (Watts et al., 2014; Rasmussen et al., 2017b; Condello et al., 2018) that were even maintained after multiple passages (Watts et al., 2014).

## Spreading

The induction of Aβ plaque pathology as consequence of exogenous seeding appears initially in the proximity to the injection site, where the highest concentration of seeds due to administration has been assumed (Kane et al., 2000; Walker et al., 2002; Meyer-Luehmann et al., 2006; Eisele et al., 2009; Hamaguchi et al., 2012; Ye et al., 2015b). Within the first day, injected material is still measurable before the Aβ signal usually becomes immunohistochemically undetectable for several months (Ye et al., 2015b). Importantly, seed-induced Aβ deposits present after several months of incubation were evidently not the injectate itself (Kane et al., 2000; Meyer-Luehmann et al., 2006; Eisele et al., 2009). Moreover, the diffusion of the injected Aβ material away from the injection site within a 7 day time-frame revealed that the initial Aβ distribution pattern in the affected regions resembled the pattern of seeded aggregation appearing 5 months after injection (Walker et al., 2002). Although seed-induced Aβ deposition close to the injection site has been shown to increase over time, it expands also to more distant and axonally interconnected brain regions (Kane et al., 2000; Walker et al., 2002; Meyer-Luehmann et al., 2006; Hamaguchi et al., 2012; Rönnbäck et al., 2012; Ye et al., 2015b), suggesting that neuronal pathways are essential for the trafficking of Aβ seeds through the brain. It was further postulated that Aβ was traveling non-randomly and depositing mostly along structures corresponding to the limbic connectome (Ye et al., 2015b). So far, there is no evidence for active transport of Aβ along neurons *in vivo*, even though it is thought to be a plausible mechanism for the spreading of Aβ pathology based on results from *in vitro* studies (Nath et al., 2012; Domert et al., 2014; Song et al., 2014; Brahic et al., 2016). Furthermore, the occurrence of intraneuronal Aβ in mice, as well as human brains, supports the hypothesis of neuronal involvement in Aβ pathology dissemination (Gouras et al., 2000; LaFerla et al., 2007). Most likely the mechanism responsible for the distribution of seeded Aβ deposits is a combination of both, passive diffusion and active transport mechanisms (Eisele and Duyckaerts, 2016). The exact cellular mechanisms involved in spreading of Aβ have not yet been elucidated. Recent studies suggested the involvement of endosomes/lysosomes or intracellular assemblies of Aβ to be crucial factors (Hu et al., 2009; Marzesco et al., 2016).

Since the transmission of Aβ seeds resembles a prion-like mechanism, the systemic routes relevant for transmission of prion diseases from periphery to CNS were also taken into account (Blättler et al., 1997; Mabbott and MacPherson, 2006; Aguzzi et al., 2008). Induction of Aβ pathology by means of oral, intraocular or intranasal routes was excluded due to absent Aβ seeding in inoculated APP-tg mice (Eisele et al., 2009). However, intraperitoneal (Eisele et al., 2010, 2014) or intravenous (Burwinkel et al., 2018) administration of Aβ-rich brain extracts induced intracerebral β-amyloidosis especially in the walls of the blood vessels in form of cerebral amyloid angiopathy (CAA). In contrast to CAA, Aβ aggregates become manifest in amyloid plaques in the brain parenchyma (Thal et al., 2015). CAA is an age-related vessel disorder that can occur in the brain of AD patients as well as non-demented elderly people and is associated with vascular dementia (Thal et al., 2012, 2015). The occurrence of CAA after administration of Aβ-rich extracts in spatially different brain regions indicates as well a role for the vascular system (Meyer-Luehmann et al., 2006; Eisele et al., 2009) or perivascular drainage channels (Weller et al., 1998; Thal et al., 2007) as possible propagation route for Aβ seeds. Whether parenchymal and vascular seed-induced Aβ deposits are generated by the same or *via* different pathways is still unknown. Nevertheless, these results provided sufficient evidence for the spread of Aβ seeds possibly *via* vascular routes. Transport of Aβ from the periphery to the brain by immune cells such as macrophages was also proposed (Eisele et al., 2014; Cintron et al., 2015), but the precise mechanisms remained unclear.

## Cross-Seeding

Several studies have proven the overlap of different neuropathological lesions such as neurofibrillary tangles (NFTs; tau), Lewy bodies (α-synuclein) or prions with Aβ pathology in brains of patients with neurodegenerative diseases like AD, PD, Dementia with Lewy Bodies (DLB) and Creutzfeldt-Jakob disease (CJD) (McKeith et al., 1996; Braak and Braak, 1997; Hamilton, 2000; McKeith, 2000, 2006; Ferrer et al., 2001; Tsuchiya et al., 2004; Debatin et al., 2008; Hyman et al., 2012; Jaunmuktane et al., 2015). *In vitro* and *in vivo* studies have demonstrated that tau, α-synuclein and prion proteins can interact with Aβ and thus influence the onset and course of the respective disease (Hamilton, 2000; McKeith, 2000; Götz et al., 2001; Lewis et al., 2001; Masliah et al., 2001; Tsigelny et al., 2008; Lasagna-Reeves et al., 2010; Morales et al., 2010).

Since several years, there has been a debate on the interaction between Aβ peptide and tau protein and its influence on the pathogenesis of AD. Both pathologies, amyloid plaques and NFTs containing hyperphosphorylated tau, are necessary for the accurate diagnosis of AD (Hyman et al., 2012). Tau pathology was also shown to be directly inducible *in vivo* in wild-type and tau tg mice after injections of tau-containing brain extracts or synthetic tau fibrils (Clavaguera et al., 2009, 2013; Lasagna-Reeves et al., 2012; Iba et al., 2013; Guo et al., 2016). According to the amyloid cascade hypothesis, amyloid deposition precedes NFT formation and posits that changes in amyloid-β lead to widespread tau pathology (Hardy and Selkoe, 2002). Early results from *in vitro* studies gave already a hint on the potency of Aβ to induce phosphorylation and aggregation of tau (Busciglio et al., 1995; Ferrari et al., 2003; Pennanen and Götz, 2005; Lasagna-Reeves et al., 2010). Infusion of synthetic Aβ fibrils, pre-aggregated Aβ or Aβ-rich extracts into the hippocampus of tau-transgenic (tau-tg) mice indeed resulted in enhanced NFT formation (Götz et al., 2001; Bolmont et al., 2007; Vasconcelos et al., 2016), similar to the effects seen in double-transgenic mice exhibiting both Aβ and tau pathology (Lewis et al., 2001; Pooler et al., 2015). Injections of human derived-tau in mice with a high Aβ plaque load developed enhanced induced tau pathology, suggesting that Aβ plaques might trigger the propagation of tau (He et al., 2018).

Furthermore, in about 50% of AD patients the presence of α-synuclein aggregates has been verified (Hamilton, 2000; Uchikado et al., 2006). Previous studies have implicated a direct interaction of Aβ and α-synuclein (Tsigelny et al., 2008). It has also been demonstrated that Aβ can trigger α-synuclein polymerization *in vitro* and overexpression of human APP/Aβ fostered the accumulation of α-synuclein and associated disease phenotype *in vivo* (Paik et al., 1998; Masliah et al., 2001). Besides, α-synuclein has been identified as a major protein accumulating in neurites in APP-tg mice, implying that Aβ might be causal for this aggregation (Yang et al., 2000). Surprisingly, APP-tg mice intracerebrally injected with α-synuclein-derived extract were devoid of seeded Aβ deposition, suggesting that α-synuclein is not able to cross-seed Aβ plaques *in vivo* (Bachhuber et al., 2015). Instead, the presence of α-synuclein even hampered amyloid plaque formation in APP-tg mice (Bachhuber et al., 2015). Moreover, brains of APP-tg mice lacking α-synuclein exhibited a significant increase in Aβ plaque load, suggesting as well a suppressive role of α-synuclein on the progression of Aβ plaque pathology (Kallhoff et al., 2007).

Finally, a role for prion protein to interact with Aβ aggregates has also been proposed. Intraperitoneal injection of prion proteins into APP-tg mice seemed to promote mature Aβ plaque formation and prion pathology was enhanced in presence of Aβ (Schwarze-Eicker et al., 2005; Morales et al., 2010). However, no cross-seeding was observed after intracerebral inoculation of infectious prions into the hippocampus of APP-tg mice (Rasmussen et al., 2018).

## Conclusion

**Figure 1:**
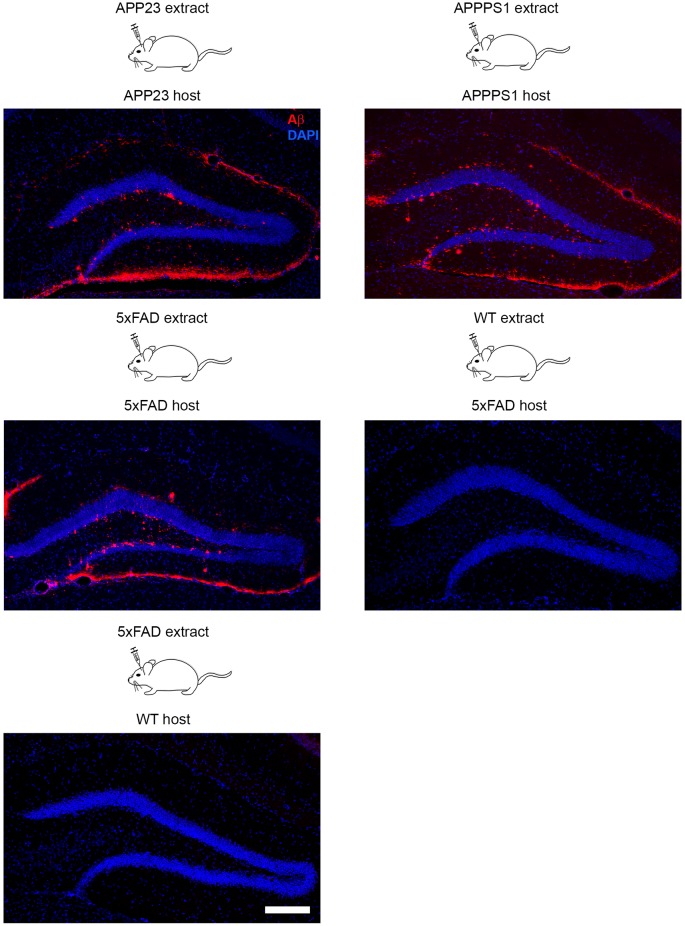
Intracerebral inoculation of brain extracts from APP23, APPPS1 and 5xFAD mice into the hippocampus of young APP-transgenic (APP-tg) host mice with genetically identical background induced seeding of Aβ pathology. In contrast, infusion of brain homogenate from a wild-type mouse into a 5xFAD tg host and vice versa failed to induce cerebral β-amyloidosis due to the lack of Aβ either in the brain extract or host animal. Fluorescence microscopy of Aβ (6E10, red) and cells (DAPI, blue). Scale bar represents 100 μm.

The seeding model of AD pathology in mice is a widely used tool to study plaque formation *in vivo* at its very early stage and within a defined time period (Kane et al., 2000; Meyer-Luehmann et al., 2006). In general it is a very robust model that was applied in many different APP-tg mouse models (Tg2576, APP/PS1, APP23, 5xFAD) with only slight variation with regard to the onset of seeding (first sign of seed-induced Aβ deposits after intracerebral inoculations) and affected brain areas (Kane et al., 2000; Meyer-Luehmann et al., 2006; Duran-Aniotz et al., 2014; Ziegler-Waldkirch et al., 2018) (Figure 1). Although seeding experiments were performed mainly in the hippocampus, the induction of Aβ deposits was additionally demonstrated in several different brain areas such as parietal cortex, striatum or olfactory bulb (Eisele et al., 2009). The greatest advantage of this model is that the accelerated Aβ plaque formation reduces the incubation time (Meyer-Luehmann et al., 2006). Furthermore, the age of the newborn plaques is easily determinable due to the predictability of the model. Characterization of seed-induced Aβ deposition and its effect on the environment at the injection side as well as areas affected by spreading of Aβ seeds can be studied as well. Recently, the consequence of Aβ seeding on adult neurogenesis and cell death was demonstrated (Ziegler-Waldkirch et al., 2018), supporting the notion that seed-induced Aβ deposits may also be a source of toxicity. Future experiments will need to test the consequences of Aβ seeding on other cell types and unravel in detail the origin of Aβ-mediated toxicity. Finally, *in vivo* seeding as a model can assist to identify factors impacting or accelerating AD progression such as microglia-derived ASC specks (Venegas et al., 2017) or the Trem2 receptor on microglia (Parhizkar et al., 2019) that might in turn contribute to the development of appropriate AD treatment.

## Author Contributions

MF and MM-L wrote, read and approved the final manuscript.

## Conflict of Interest

The authors declare that the research was conducted in the absence of any commercial or financial relationships that could be construed as a potential conflict of interest.

## References

[B1] Aguzzi A. Sigurdson C. Heikenwaelder M. (2008). Molecular mechanisms of prion pathogenesis. Annu. Rev. Pathol. 3, 11–40. 10.1146/annurev.pathmechdis.3.121806.154326 18233951

[B2] Bachhuber T. Katzmarski N. McCarter J. F. Loreth D. Tahirovic S. Kamp F.. (2015). Inhibition of amyloid-β plaque formation by α-synuclein. Nat. Med. 21, 802–807. 10.1038/nm.3885 26099047

[B3] Baker H. F. Ridley R. M. Duchen L. W. Crow T. J. Bruton C. J. (1993). Evidence for the experimental transmission of cerebral β-amyloidosis to primates. Int. J. Exp. Pathol. 74, 441–454. 8217779 PMC2002177

[B4] Baker H. F. Ridley R. M. Duchen L. W. Crow T. J. Bruton C. J. (1994). Induction of β(A4)-amyloid in primates by injection of Alzheimer’s disease brain homogenate. Mol. Neurobiol. 8, 25–39. 10.1007/BF02778005 8086126

[B5] Blättler T. Brandner S. Raeber A. J. Klein M. A. Voigtländer T. Weissmann C.. (1997). PrP-expressing tissue required for transfer of scrapie infectivity from spleen to brain. Nature 389, 69–73. 10.1038/37981 9288968

[B6] Bolmont T. Clavaguera F. Meyer-Luehmann M. Herzig M. C. Radde R. Staufenbiel M.. (2007). Induction of tau pathology by intracerebral infusion of amyloid-β-containing brain extract and by amyloid-β deposition in APP x Tau transgenic mice. Am. J. Pathol. 171, 2012–2020. 10.2353/ajpath.2007.070403 18055549 PMC2111123

[B7] Braak H. Braak E. (1997). Frequency of stages of Alzheimer-related lesions in different age categories. Neurobiol. Aging 18, 351–357. 10.1016/s0197-4580(97)00056-0 9330961

[B8] Brahic M. Bousset L. Bieri G. Melki R. Gitler A. D. (2016). Axonal transport and secretion of fibrillar forms of α-synuclein, Aβ42 peptide and HTTExon 1. Acta Neuropathol. 131, 539–548. 10.1007/s00401-016-1538-0 26820848 PMC4789229

[B9] Burwinkel M. Lutzenberger M. Heppner F. L. Schulz-Schaeffer W. Baier M. (2018). Intravenous injection of β-amyloid seeds promotes cerebral amyloid angiopathy (CAA). Acta Neuropathol. Commun. 6:23. 10.1186/s40478-018-0511-7 29506560 PMC5836327

[B10] Busciglio J. Lorenzo A. Yeh J. Yankner B. A. (1995). β-amyloid fibrils induce tau phosphorylation and loss of microtubule binding. Neuron 14, 879–888. 10.1016/0896-6273(95)90232-5 7718249

[B11] Cintron A. F. Dalal N. V. Dooyema J. Betarbet R. Walker L. C. (2015). Transport of cargo from periphery to brain by circulating monocytes. Brain Res. 1622, 328–338. 10.1016/j.brainres.2015.06.047 26168900 PMC4642726

[B12] Clavaguera F. Akatsu H. Fraser G. Crowther R. A. Frank S. Hench J.. (2013). Brain homogenates from human tauopathies induce tau inclusions in mouse brain. Proc. Natl. Acad. Sci. U S A 110, 9535–9540. 10.1073/pnas.1301175110 23690619 PMC3677441

[B13] Clavaguera F. Bolmont T. Crowther R. A. Abramowski D. Frank S. Probst A.. (2009). Transmission and spreading of tauopathy in transgenic mouse brain. Nat. Cell Biol. 11, 909–913. 10.1038/ncb1901 19503072 PMC2726961

[B14] Cleary J. P. Walsh D. M. Hofmeister J. J. Shankar G. M. Kuskowski M. A. Selkoe D. J.. (2005). Natural oligomers of the amyloid-β protein specifically disrupt cognitive function. Nat. Neurosci. 8, 79–84. 10.1038/nn1372 15608634

[B15] Cohen E. Paulsson J. F. Blinder P. Burstyn-Cohen T. Du D. Estepa G.. (2009). Reduced IGF-1 signaling delays age-associated proteotoxicity in mice. Cell 139, 1157–1169. 10.1016/j.cell.2009.11.014 20005808 PMC3017511

[B16] Cohen S. I. A. Linse S. Luheshi L. M. Hellstrand E. White D. A. Rajah L.. (2013). Proliferation of amyloid-β42 aggregates occurs through a secondary nucleation mechanism. Proc. Natl. Acad. Sci. U S A 110, 9758–9763. 10.1073/pnas.1218402110 23703910 PMC3683769

[B17] Condello C. Lemmin T. Stöhr J. Nick M. Wu Y. Maxwell A. M.. (2018). Structural heterogeneity and intersubject variability of Aβ in familial and sporadic Alzheimer’s disease. Proc. Natl. Acad. Sci. U S A 115, E782–E791. 10.1073/pnas.1714966115 29311311 PMC5789926

[B18] Debatin L. Streffer J. Geissen M. Matschke J. Aguzzi A. Glatzel M. (2008). Association between deposition of β-amyloid and pathological prion protein in sporadic Creutzfeldt-Jakob disease. Neurodegener. Dis. 5, 347–354. 10.1159/000121389 18349519

[B19] Domert J. Rao S. B. Agholme L. Brorsson A.-C. Marcusson J. Hallbeck M.. (2014). Spreading of amyloid-β peptides *via* neuritic cell-to-cell transfer is dependent on insufficient cellular clearance. Neurobiol. Dis. 65, 82–92. 10.1016/j.nbd.2013.12.019 24412310

[B20] Duran-Aniotz C. Morales R. Moreno-Gonzalez I. Hu P. P. Fedynyshyn J. Soto C. (2014). Aggregate-depleted brain fails to induce Aβ deposition in a mouse model of Alzheimer’s disease. PLoS One 9:e89014. 10.1371/journal.pone.0089014 24533166 PMC3923072

[B22] Eisele Y. S. Bolmont T. Heikenwalder M. Langer F. Jacobson L. H. Yan Z.-X.. (2009). Induction of cerebral β-amyloidosis: intracerebral versus systemic Aβ inoculation. Proc. Natl. Acad. Sci. U S A 106, 12926–12931. 10.1073/pnas.0903200106 19622727 PMC2722323

[B21] Eisele Y. S. Duyckaerts C. (2016). Propagation of A*ß*pathology: hypotheses, discoveries, and yet unresolved questions from experimental and human brain studies. Acta Neuropathol. 131, 5–25. 10.1007/s00401-015-1516-y 26715565

[B23] Eisele Y. S. Fritschi S. K. Hamaguchi T. Obermüller U. Füger P. Skodras A.. (2014). Multiple factors contribute to the peripheral induction of cerebral β-amyloidosis. J. Neurosci. 34, 10264–10273. 10.1523/JNEUROSCI.1608-14.2014 25080588 PMC6608275

[B24] Eisele Y. S. Obermüller U. Heilbronner G. Baumann F. Kaeser S. A. Wolburg H.. (2010). Peripherally applied Aβ-containing inoculates induce cerebral β-amyloidosis. Science 330, 980–982. 10.1126/science.1194516 20966215 PMC3233904

[B25] Eisenberg D. Jucker M. (2012). The amyloid state of proteins in human diseases. Cell 148, 1188–1203. 10.1016/j.cell.2012.02.022 22424229 PMC3353745

[B26] Falsig J. Nilsson K. P. Knowles T. P. J. Aguzzi A. (2008). Chemical and biophysical insights into the propagation of prion strains. HFSP J. 2, 332–341. 10.2976/1.2990786 19436493 PMC2645583

[B27] Fändrich M. Meinhardt J. Grigorieff N. (2009). Structural polymorphism of Alzheimer Aβ and other amyloid fibrils. Prion 3, 89–93. 10.4161/pri.3.2.8859 19597329 PMC2712605

[B28] Ferrari A. Hoerndli F. Baechi T. Nitsch R. M. Götz J. (2003). β-Amyloid induces paired helical filament-like tau filaments in tissue culture. J. Biol. Chem. 278, 40162–40168. 10.1074/jbc.M308243200 12893817

[B29] Ferrer I. Blanco R. Carmona M. Puig B. Ribera R. Rey M. J.. (2001). Prion protein expression in senile plaques in Alzheimer’s disease. Acta Neuropathol. 101, 49–56. 10.1007/s004010000271 11194941

[B30] Fritschi S. K. Cintron A. Ye L. Mahler J. Bühler A. Baumann F.. (2014a). Aβ seeds resist inactivation by formaldehyde. Acta Neuropathol. 128, 477–484. 10.1007/s00401-014-1339-2 25193240 PMC4169116

[B31] Fritschi S. K. Langer F. Kaeser S. A. Maia L. F. Portelius E. Pinotsi D.. (2014b). Highly potent soluble amyloid-β seeds in human Alzheimer brain but not cerebrospinal fluid. Brain J. Neurol. 137, 2909–2915. 10.1093/brain/awu255 25212850 PMC5367518

[B32] Gajdusek D. C. (1977). Unconventional viruses and the origin and disappearance of kuru. Science 197, 943–960. 10.1126/science.142303 142303

[B33] Gajdusek D. C. Gibbs C. J. Alpers M. (1966). Experimental transmission of a kuru-like syndrome to chimpanzees. Nature 209, 794–796. 10.1038/209794a0 5922150

[B34] Gibbs C. J. Gajdusek D. C. Asher D. M. Alpers M. P. Beck E. Daniel P. M.. (1968). Creutzfeldt-Jakob disease (spongiform encephalopathy): transmission to the chimpanzee. Science 161, 388–389. 10.1126/science.161.3839.388 5661299

[B35] Glenner G. G. Wong C. W. (1984). Alzheimer’s disease: initial report of the purification and characterization of a novel cerebrovascular amyloid protein. Biochem. Biophys. Res. Commun. 120, 885–890. 10.1016/s0006-291x(84)80190-4 6375662

[B36] Götz J. Chen F. van Dorpe J. Nitsch R. M. (2001). Formation of neurofibrillary tangles in P301l tau transgenic mice induced by Aβ 42 fibrils. Science 293, 1491–1495. 10.1126/science.1062097 11520988

[B37] Goudsmit J. Morrow C. H. Asher D. M. Yanagihara R. T. Masters C. L. Gibbs C. J.. (1980). Evidence for and against the transmissibility of Alzheimer disease. Neurology 30, 945–950. 10.1212/wnl.30.9.945 6775247

[B38] Gouras G. K. Tsai J. Naslund J. Vincent B. Edgar M. Checler F.. (2000). Intraneuronal Aβ42 accumulation in human brain. Am. J. Pathol. 156, 15–20. 10.1016/s0002-9440(10)64700-1 10623648 PMC1868613

[B39] Guo J. L. Narasimhan S. Changolkar L. He Z. Stieber A. Zhang B.. (2016). Unique pathological tau conformers from Alzheimer’s brains transmit tau pathology in nontransgenic mice. J. Exp. Med. 213, 2635–2654. 10.1084/jem.20160833 27810929 PMC5110027

[B40] Hadlow W. J. Prusiner S. B. Kennedy R. C. Race R. E. (1980). Brain tissue from persons dying of Creutzfeldt-Jakob disease causes scrapie-like encephalopathy in goats. Ann. Neurol. 8, 628–632. 10.1002/ana.410080615 7011169

[B41] Hamaguchi T. Eisele Y. S. Varvel N. H. Lamb B. T. Walker L. C. Jucker M. (2012). The presence of Aβ, seeds and not age *per se*, is critical to the initiation of Aβ deposition in the brain. Acta Neuropathol. 123, 31–37. 10.1007/s00401-011-0912-1 22101366 PMC3297471

[B42] Hamilton R. L. (2000). Lewy bodies in Alzheimer’s disease: a neuropathological review of 145 cases using α-synuclein immunohistochemistry. Brain Pathol. 10, 378–384. 10.1111/j.1750-3639.2000.tb00269.x 10885656 PMC8098522

[B43] Hardy J. Selkoe D. J. (2002). The amyloid hypothesis of Alzheimer’s disease: progress and problems on the road to therapeutics. Science 297, 353–356. 10.1126/science.1072994 12130773

[B44] Harper J. D. Lansbury P. T. Jr. (1997). Models of amyloid seeding in Alzheimer’s disease and scrapie: mechanistic truths and physiological consequences of the time-dependent solubility of amyloid proteins. Annu. Rev. Biochem. 66, 385–407. 10.1146/annurev.biochem.66.1.385 9242912

[B45] Harper J. D. Wong S. S. Lieber C. M. Lansbury P. T. (1997). Observation of metastable Aβ amyloid protofibrils by atomic force microscopy. Chem. Biol. 4, 119–125. 10.1016/s1074-5521(97)90255-6 9190286

[B46] He Z. Guo J. L. McBride J. D. Narasimhan S. Kim H. Changolkar L.. (2018). Amyloid-β plaques enhance Alzheimer’s brain tau-seeded pathologies by facilitating neuritic plaque tau aggregation. Nat. Med. 24, 29–38. 10.1038/nm.4443 29200205 PMC5760353

[B47] Heilbronner G. Eisele Y. S. Langer F. Kaeser S. A. Novotny R. Nagarathinam A.. (2013). Seeded strain-like transmission of β-amyloid morphotypes in APP transgenic mice. EMBO Rep. 14, 1017–1022. 10.1038/embor.2013.137 23999102 PMC3818077

[B48] Heuer E. Rosen R. F. Cintron A. Walker L. C. (2012). Nonhuman primate models of Alzheimer-like cerebral proteopathy. Curr. Pharm. Des. 18, 1159–1169. 10.2174/138161212799315885 22288403 PMC3381739

[B49] Hu X. Crick S. L. Bu G. Frieden C. Pappu R. V. Lee J.-M. (2009). Amyloid seeds formed by cellular uptake, concentration, and aggregation of the amyloid-β peptide. Proc. Natl. Acad. Sci. U S A 106, 20324–20329. 10.1073/pnas.0911281106 19910533 PMC2787156

[B50] Hyman B. T. Phelps C. H. Beach T. G. Bigio E. H. Cairns N. J. Carrillo M. C.. (2012). National institute on aging-Alzheimer’s association guidelines for the neuropathologic assessment of Alzheimer’s disease. Alzheimers Dement. 8, 1–13. 10.1016/j.jalz.2011.10.007 22265587 PMC3266529

[B51] Iba M. Guo J. L. McBride J. D. Zhang B. Trojanowski J. Q. Lee V. M.-Y. (2013). Synthetic tau fibrils mediate transmission of neurofibrillary tangles in a transgenic mouse model of Alzheimer’s-like tauopathy. J. Neurosci. 33, 1024–1037. 10.1523/JNEUROSCI.2642-12.2013 23325240 PMC3575082

[B54] Jarrett J. T. Berger E. P. Lansbury P. T. Jr. (1993). The carboxy terminus of the .beta. amyloid protein is critical for the seeding of amyloid formation: implications for the pathogenesis of Alzheimer’s disease. Biochemistry 32, 4693–4697. 10.1021/bi00069a001 8490014

[B52] Jarrett J. T. Lansbury P. T. Jr. (1992). Amyloid fibril formation requires a chemically discriminating nucleation event: studies of an amyloidogenic sequence from the bacterial protein OsmB. Biochemistry 31, 12345–12352. 10.1021/bi00164a008 1463722

[B53] Jarrett J. T. Lansbury P. T. Jr. (1993). Seeding “one-dimensional crystallization” of amyloid: a pathogenic mechanism in Alzheimer’s disease and scrapie? Cell 73, 1055–1058. 10.1016/0092-8674(93)90635-4 8513491

[B55] Jaunmuktane Z. Mead S. Ellis M. Wadsworth J. D. F. Nicoll A. J. Kenny J.. (2015). Evidence for human transmission of amyloid-β pathology and cerebral amyloid angiopathy. Nature 525, 247–250. 10.1038/nature15369 26354483

[B56] Kallhoff V. Peethumnongsin E. Zheng H. (2007). Lack of α-synuclein increases amyloid plaque accumulation in a transgenic mouse model of Alzheimer’s disease. Mol. Neurodegener. 2:6. 10.1186/1750-1326-2-6 17367539 PMC1832188

[B57] Kane M. D. Lipinski W. J. Callahan M. J. Bian F. Durham R. A. Schwarz R. D.. (2000). Evidence for seeding of β -amyloid by intracerebral infusion of Alzheimer brain extracts in β -amyloid precursor protein-transgenic mice. J. Neurosci. 20, 3606–3611. 10.1523/JNEUROSCI.20-10-03606.2000 10804202 PMC6772682

[B58] Katzmarski N. Ziegler-Waldkirch S. Scheffler N. Witt C. Abou-Ajram C. Nuscher B.. (2019). Aβ oligomers trigger and accelerate Aβ seeding. Brain Pathol. [Epub ahead of print]. 10.1111/bpa.12734 31099449 PMC6916291

[B59] Knowles T. P. J. Waudby C. A. Devlin G. L. Cohen S. I. A. Aguzzi A. Vendruscolo M.. (2009). An analytical solution to the kinetics of breakable filament assembly. Science 326, 1533–1537. 10.1126/science.1178250 20007899

[B60] LaFerla F. M. Green K. N. Oddo S. (2007). Intracellular amyloid-β in Alzheimer’s disease. Nat. Rev. Neurosci. 8, 499–509. 10.1038/nrn2168 17551515

[B61] Lambert M. P. Barlow A. K. Chromy B. A. Edwards C. Freed R. Liosatos M.. (1998). Diffusible, nonfibrillar ligands derived from Aβ1–42 are potent central nervous system neurotoxins. Proc. Natl. Acad. Sci. U S A 95, 6448–6453. 10.1073/pnas.95.11.6448 9600986 PMC27787

[B62] Langer F. Eisele Y. S. Fritschi S. K. Staufenbiel M. Walker L. C. Jucker M. (2011). Soluble Aβ seeds are potent inducers of cerebral β-amyloid deposition. J. Neurosci. 31, 14488–14495. 10.1523/JNEUROSCI.3088-11.2011 21994365 PMC3229270

[B63] Lasagna-Reeves C. A. Castillo-Carranza D. L. Guerrero-Muoz M. J. Jackson G. R. Kayed R. (2010). Preparation and characterization of neurotoxic tau oligomers. Biochemistry 49, 10039–10041. 10.1021/bi1016233 21047142

[B64] Lasagna-Reeves C. A. Castillo-Carranza D. L. Sengupta U. Guerrero-Munoz M. J. Kiritoshi T. Neugebauer V.. (2012). Alzheimer brain-derived tau oligomers propagate pathology from endogenous tau. Sci. Rep. 2:700. 10.1038/srep00700 23050084 PMC3463004

[B65] Lesné S. Koh M. T. Kotilinek L. Kayed R. Glabe C. G. Yang A.. (2006). A specific amyloid-β protein assembly in the brain impairs memory. Nature 440, 352–357. 10.1038/nature04533 16541076

[B66] Levine H. III. Walker L. C. (2010). Molecular polymorphism of Aβ in Alzheimer’s disease. Neurobiol. Aging 31, 542–548. 10.1016/j.neurobiolaging.2008.05.026 18619711 PMC2842206

[B67] Lewis J. Dickson D. W. Lin W. L. Chisholm L. Corral A. Jones G.. (2001). Enhanced neurofibrillary degeneration in transgenic mice expressing mutant tau and APP. Science 293, 1487–1491. 10.1126/science.1058189 11520987

[B68] Lomakin A. Chung D. S. Benedek G. B. Kirschner D. A. Teplow D. B. (1996). On the nucleation and growth of amyloid β-protein fibrils: detection of nuclei and quantitation of rate constants. Proc. Natl. Acad. Sci. U S A 93, 1125–1129. 10.1073/pnas.93.3.1125 8577726 PMC40042

[B69] Lomakin A. Teplow D. B. Kirschner D. A. Benedek G. B. (1997). Kinetic theory of fibrillogenesis of amyloid β-protein. Proc. Natl. Acad. Sci. U S A 94, 7942–7947. 10.1073/pnas.94.15.7942 9223292 PMC21534

[B70] Lu J.-X. Qiang W. Yau W.-M. Schwieters C. D. Meredith S. C. Tycko R. (2013). Molecular structure of β-amyloid fibrils in Alzheimer’s disease brain tissue. Cell 154, 1257–1268. 10.1016/j.cell.2013.08.035 24034249 PMC3814033

[B71] Luk K. C. Kehm V. Carroll J. Zhang B. O’Brien P. Trojanowski J. Q.. (2012). Pathological α-synuclein transmission initiates Parkinson-like neurodegeneration in nontransgenic mice. Science 338, 949–953. 10.1126/science.1227157 23161999 PMC3552321

[B72] Mabbott N. A. MacPherson G. G. (2006). Prions and their lethal journey to the brain. Nat. Rev. Microbiol. 4, 201–211. 10.1038/nrmicro1346 16462753

[B73] Maclean C. J. Baker H. F. Ridley R. M. Mori H. (2000). Naturally occurring and experimentally induced β-amyloid deposits in the brains of marmosets (*Callithrix jacchus*). J. Neural Transm. 107, 799–814. 10.1007/s007020070060 11005545

[B74] Manuelidis E. E. Manuelidis L. (1991). “Search for a transmissible agent in Alzheimer’s disease: studies of human buffy coat,” in Transmissible Spongiform Encephalopathies: Scrapie, BSE and Related Human Disorders Current Topics in Microbiology and Immunology, ed. Chesebro B. W. (Berlin, Heidelberg: Springer Berlin Heidelberg), 275–280. 10.1007/978-3-642-76540-7_16 1810711

[B75] Marzesco A.-M. Flötenmeyer M. Bühler A. Obermüller U. Staufenbiel M. Jucker M.. (2016). Highly potent intracellular membrane-associated Aβ seeds. Sci. Rep. 6:28125. 10.1038/srep28125 27311744 PMC4911570

[B76] Masliah E. Rockenstein E. Veinbergs I. Sagara Y. Mallory M. Hashimoto M.. (2001). β-amyloid peptides enhance α-synuclein accumulation and neuronal deficits in a transgenic mouse model linking Alzheimer’s disease and Parkinson’s disease. Proc. Natl. Acad. Sci. U S A 98, 12245–12250. 10.1073/pnas.211412398 11572944 PMC59799

[B77] McKeith I. G. (2000). Spectrum of Parkinson’s disease, Parkinson’s dementia, and Lewy body dementia. Neurol. Clin. 18, 865–902. 10.1016/S0733-8619(05)70230-9 11072265

[B78] McKeith I. G. (2006). Consensus guidelines for the clinical and pathologic diagnosis of dementia with Lewy bodies (DLB): report of the Consortium on DLB International Workshop. J. Alzheimers Dis. 9, 417–423. 10.3233/jad-2006-9s347 16914880

[B79] McKeith I. G. Galasko D. Kosaka K. Perry E. K. Dickson D. W. Hansen L. A.. (1996). Consensus guidelines for the clinical and pathologic diagnosis of dementia with Lewy bodies (DLB): report of the consortium on DLB international workshop. Neurology 47, 1113–1124. 10.1212/wnl.47.5.1113 8909416

[B80] Meinhardt J. Sachse C. Hortschansky P. Grigorieff N. Fändrich M. (2009). Aβ(1–40) fibril polymorphism implies diverse interaction patterns in amyloid fibrils. J. Mol. Biol. 386, 869–877. 10.1016/j.jmb.2008.11.005 19038266 PMC6760659

[B81] Meyer-Luehmann M. Coomaraswamy J. Bolmont T. Kaeser S. Schaefer C. Kilger E.. (2006). Exogenous induction of cerebral β-amyloidogenesis is governed by agent and host. Science 313, 1781–1784. 10.1126/science.1131864 16990547

[B82] Meyer-Luehmann M. Mielke M. Spires-Jones T. L. Stoothoff W. Jones P. Bacskai B. J.. (2009). A reporter of local dendritic translocation shows plaque- related loss of neural system function in APP-transgenic mice. J. Neurosci. 29, 12636–12640. 10.1523/JNEUROSCI.1948-09.2009 19812338 PMC2789808

[B83] Meyer-Luehmann M. Spires-Jones T. L. Prada C. Garcia-Alloza M. de Calignon A. Rozkalne A.. (2008). Rapid appearance and local toxicity of amyloid-β plaques in a mouse model of Alzheimer’s disease. Nature 451, 720–724. 10.1038/nature06616 18256671 PMC3264491

[B84] Morales R. Duran-Aniotz C. Castilla J. Estrada L. D. Soto C. (2012). *De novo* induction of amyloid-β deposition *in vivo*. Mol. Psychiatry 17, 1347–1353. 10.1038/mp.2011.120 21968933

[B85] Morales R. Estrada L. D. Diaz-Espinoza R. Morales-Scheihing D. Jara M. C. Castilla J.. (2010). Molecular cross talk between misfolded proteins in animal models of Alzheimer’s and prion diseases. J. Neurosci. 30, 4528–4535. 10.1523/JNEUROSCI.5924-09.2010 20357103 PMC2859074

[B86] Morales R. Green K. M. Soto C. (2009). Cross currents in protein misfolding disorders: interactions and therapy. CNS Neurol. Disord. Drug Targets 8, 363–371. 10.2174/187152709789541998 19702573 PMC2804467

[B87] Morales R. Moreno-Gonzalez I. Soto C. (2013). Cross-seeding of misfolded proteins: implications for etiology and pathogenesis of protein misfolding diseases. PLoS Pathog. 9:e1003537. 10.1371/journal.ppat.1003537 24068917 PMC3777858

[B88] Nath S. Agholme L. Kurudenkandy F. R. Granseth B. Marcusson J. Hallbeck M. (2012). Spreading of neurodegenerative pathology *via* neuron-to-neuron transmission of β-amyloid. J. Neurosci. 32, 8767–8777. 10.1523/JNEUROSCI.0615-12.2012 22745479 PMC6622335

[B89] Nilsson K. P. R. Aslund A. Berg I. Nyström S. Konradsson P. Herland A.. (2007). Imaging distinct conformational states of amyloid-β fibrils in Alzheimer’s disease using novel luminescent probes. ACS Chem. Biol. 2, 553–560. 10.1021/cb700116u 17672509

[B90] Novotny R. Langer F. Mahler J. Skodras A. Vlachos A. Wegenast-Braun B. M.. (2016). Conversion of synthetic Aβ to *in vivo* active seeds and amyloid plaque formation in a hippocampal slice culture model. J. Neurosci. 36, 5084–5093. 10.1523/JNEUROSCI.0258-16.2016 27147660 PMC6601857

[B300] Otvos L. Jr. Szendrei G. I. Lee V. M. Mantsch H. H. (1993). Human and rodent Alzheimer beta-amyloid peptides acquire distinct conformations in membrane-mimicking solvents. Eur. J. Biochem 211, 249–257. 10.1111/j.1432-1033.1993.tb19893.x 8425535

[B91] Paik S. R. Lee J. H. Kim D. H. Chang C. S. Kim Y. S. (1998). Self-oligomerization of NACP, the precursor protein of the non-amyloid β/A4 protein (Aβ) component of Alzheimer’s disease amyloid, observed in the presence of a C-terminal Aβ fragment (residues 25–35). FEBS Lett. 421, 73–76. 10.1016/s0014-5793(97)01537-8 9462843

[B92] Paravastu A. K. Leapman R. D. Yau W.-M. Tycko R. (2008). Molecular structural basis for polymorphism in Alzheimer’s β-amyloid fibrils. Proc. Natl. Acad. Sci. U S A 105, 18349–18354. 10.1073/pnas.0806270105 19015532 PMC2587602

[B93] Paravastu A. K. Qahwash I. Leapman R. D. Meredith S. C. Tycko R. (2009). Seeded growth of β-amyloid fibrils from Alzheimer’s brain-derived fibrils produces a distinct fibril structure. Proc. Natl. Acad. Sci. U S A 106, 7443–7448. 10.1073/pnas.0812033106 19376973 PMC2678625

[B94] Parhizkar S. Arzberger T. Brendel M. Kleinberger G. Deussing M. Focke C.. (2019). Loss of TREM2 function increases amyloid seeding but reduces plaque-associated ApoE. Nat. Neurosci. 22, 191–204. 10.1038/s41593-018-0296-9 30617257 PMC6417433

[B95] Pennanen L. Götz J. (2005). Different tau epitopes define Aβ42-mediated tau insolubility. Biochem. Biophys. Res. Commun. 337, 1097–1101. 10.1016/j.bbrc.2005.09.168 16226718

[B96] Petkova A. T. Leapman R. D. Guo Z. Yau W.-M. Mattson M. P. Tycko R. (2005). Self-propagating, molecular-level polymorphism in Alzheimer’s β-amyloid fibrils. Science 307, 262–265. 10.1126/science.1105850 15653506

[B97] Piccini A. Russo C. Gliozzi A. Relini A. Vitali A. Borghi R.. (2005). β-amyloid is different in normal aging and in Alzheimer disease. J. Biol. Chem. 280, 34186–34192. 10.1074/jbc.M501694200 16103127

[B98] Pooler A. M. Polydoro M. Maury E. A. Nicholls S. B. Reddy S. M. Wegmann S.. (2015). Amyloid accelerates tau propagation and toxicity in a model of early Alzheimer’s disease. Acta Neuropathol. Commun. 3:14. 10.1186/s40478-015-0199-x 25853174 PMC4371800

[B99] Portelius E. Lashley T. Westerlund A. Persson R. Fox N. C. Blennow K.. (2015). Brain amyloid-β fragment signatures in pathological ageing and Alzheimer’s disease by hybrid immunoprecipitation mass spectrometry. Neurodegener. Dis. 15, 50–57. 10.1159/000369465 25591542

[B100] Prusiner S. B. (1982). Novel proteinaceous infectious particles cause scrapie. Science 216, 136–144. 10.1126/science.6801762 6801762

[B101] Prusiner S. B. (1984). Some speculations about prions, amyloid, and Alzheimer’s disease. N. Engl. J. Med. 310, 661–663. 10.1056/nejm198403083101021 6363926

[B102] Qiang W. Yau W.-M. Lu J.-X. Collinge J. Tycko R. (2017). Structural variation in amyloid-β fibrils from Alzheimer’s disease clinical subtypes. Nature 541, 217–221. 10.1038/nature20814 28052060 PMC5233555

[B103] Rasmussen J. Jucker M. Walker L. C. (2017a). Aβ seeds and prions: how close the fit? Prion 11, 215–225. 10.1080/19336896.2017.1334029 28657440 PMC5553305

[B105] Rasmussen J. Mahler J. Beschorner N. Kaeser S. A. Häsler L. M. Baumann F.. (2017b). Amyloid polymorphisms constitute distinct clouds of conformational variants in different etiological subtypes of Alzheimer’s disease. Proc. Natl. Acad. Sci. U S A 114, 13018–13023. 10.1073/pnas.1713215114 29158413 PMC5724274

[B104] Rasmussen J. Krasemann S. Altmeppen H. Schwarz P. Schelle J. Aguzzi A.. (2018). Infectious prions do not induce Aβ deposition in an *in vivo* seeding model. Acta Neuropathol. 135, 965–967. 10.1007/s00401-018-1848-5 29663066

[B106] Ridley R. M. Baker H. F. Windle C. P. Cummings R. M. (2006). Very long term studies of the seeding of β-amyloidosis in primates. J. Neural Transm. 113, 1243–1251. 10.1007/s00702-005-0385-2 16362635

[B107] Rönnbäck A. Sagelius H. Bergstedt K. D. Näslund J. Westermark G. T. Winblad B.. (2012). Amyloid neuropathology in the single Arctic APP transgenic model affects interconnected brain regions. Neurobiol. Aging 33, 831.e11–831.e19. 10.1016/j.neurobiolaging.2011.07.012 21880397

[B108] Rosen R. F. Fritz J. J. Dooyema J. Cintron A. F. Hamaguchi T. Lah J. J.. (2012). Exogenous seeding of cerebral β-amyloid deposition in βAPP-transgenic rats. J. Neurochem. 120, 660–666. 10.1111/j.1471-4159.2011.07551.x 22017494 PMC3293176

[B109] Ruiz-Riquelme A. Lau H. H. C. Stuart E. Goczi A. N. Wang Z. Schmitt-Ulms G.. (2018). Prion-like propagation of β-amyloid aggregates in the absence of APP overexpression. Acta Neuropathol. Commun. 6:26. 10.1186/s40478-018-0529-x 29615128 PMC5883524

[B110] Schwarze-Eicker K. Keyvani K. Görtz N. Westaway D. Sachser N. Paulus W. (2005). Prion protein (PrPc) promotes β-amyloid plaque formation. Neurobiol. Aging 26, 1177–1182. 10.1016/j.neurobiolaging.2004.10.004 15917101

[B111] Shankar G. M. Li S. Mehta T. H. Garcia-Munoz A. Shepardson N. E. Smith I.. (2008). Amyloid-β protein dimers isolated directly from Alzheimer’s brains impair synaptic plasticity and memory. Nat. Med. 14, 837–842. 10.1038/nm1782 18568035 PMC2772133

[B112] Silveira J. R. Raymond G. J. Hughson A. G. Race R. E. Sim V. L. Hayes S. F.. (2005). The most infectious prion protein particles. Nature 437, 257–261. 10.1038/nature03989 16148934 PMC1513539

[B113] Song H.-L. Shim S. Kim D.-H. Won S.-H. Joo S. Kim S.. (2014). β-Amyloid is transmitted *via* neuronal connections along axonal membranes. Ann. Neurol. 75, 88–97. 10.1002/ana.24029 24114864

[B114] Spirig T. Ovchinnikova O. Vagt T. Glockshuber R. (2014). Direct evidence for self-propagation of different amyloid-β fibril conformations. Neurodegener. Dis. 14, 151–159. 10.1159/000363623 25300967

[B115] Stern E. A. Bacskai B. J. Hickey G. A. Attenello F. J. Lombardo J. A. Hyman B. T. (2004). Cortical synaptic integration *in vivo* is disrupted by amyloid-β plaques. J. Neurosci. 24, 4535–4540. 10.1523/jneurosci.0462-04.2004 15140924 PMC6729398

[B116] Stöhr J. Condello C. Watts J. C. Bloch L. Oehler A. Nick M.. (2014). Distinct synthetic Aβ prion strains producing different amyloid deposits in bigenic mice. Proc. Natl. Acad. Sci. U S A 111, 10329–10334. 10.1073/pnas.1408968111 24982137 PMC4104853

[B117] Stöhr J. Watts J. C. Mensinger Z. L. Oehler A. Grillo S. K. DeArmond S. J.. (2012). Purified and synthetic Alzheimer’s amyloid β (Aβ) prions. Proc. Natl. Acad. Sci. U S A 109, 11025–11030. 10.1073/pnas.1206555109 22711819 PMC3390876

[B118] Szaruga M. Veugelen S. Benurwar M. Lismont S. Sepulveda-Falla D. Lleo A.. (2015). Qualitative changes in human γ-secretase underlie familial Alzheimer’s disease. J. Exp. Med. 212, 2003–2013. 10.1084/jem.20150892 26481686 PMC4647268

[B119] Thal D. R. Grinberg L. T. Attems J. (2012). Vascular dementia: different forms of vessel disorders contribute to the development of dementia in the elderly brain. Exp. Gerontol. 47, 816–824. 10.1016/j.exger.2012.05.023 22705146 PMC3470831

[B120] Thal D. R. Larionov S. Abramowski D. Wiederhold K.-H. Van Dooren T. Yamaguchi H.. (2007). Occurrence and co-localization of amyloid β-protein and apolipoprotein E in perivascular drainage channels of wild-type and APP-transgenic mice. Neurobiol. Aging 28, 1221–1230. 10.1016/j.neurobiolaging.2006.05.029 16815595

[B121] Thal D. R. Walter J. Saido T. C. Fändrich M. (2015). Neuropathology and biochemistry of Aβ and its aggregates in Alzheimer’s disease. Acta Neuropathol. 129, 167–182. 10.1007/s00401-014-1375-y 25534025

[B122] Townsend M. Shankar G. M. Mehta T. Walsh D. M. Selkoe D. J. (2006). Effects of secreted oligomers of amyloid β-protein on hippocampal synaptic plasticity: a potent role for trimers. J. Physiol. 572, 477–492. 10.1113/jphysiol.2005.103754 16469784 PMC1779683

[B123] Tsai J. Grutzendler J. Duff K. Gan W.-B. (2004). Fibrillar amyloid deposition leads to local synaptic abnormalities and breakage of neuronal branches. Nat. Neurosci. 7, 1181–1183. 10.1038/nn1335 15475950

[B124] Tsigelny I. F. Crews L. Desplats P. Shaked G. M. Sharikov Y. Mizuno H.. (2008). Mechanisms of hybrid oligomer formation in the pathogenesis of combined Alzheimer’s and Parkinson’s diseases. PLoS One 3:e3135. 10.1371/journal.pone.0003135 18769546 PMC2519786

[B125] Tsuchiya K. Yagishita S. Ikeda K. Sano M. Taki K. Hashimoto K.. (2004). Coexistence of CJD and Alzheimer’s disease: an autopsy case showing typical clinical features of CJD. Neuropathology 24, 46–55. 10.1111/j.1440-1789.2003.00513.x 15068172

[B126] Uchikado H. Lin W.-L. DeLucia M. W. Dickson D. W. (2006). Alzheimer disease with amygdala Lewy bodies: a distinct form of α-synucleinopathy. J. Neuropathol. Exp. Neurol. 65, 685–697. 10.1097/01.jnen.0000225908.90052.07 16825955 PMC5706655

[B127] Vasconcelos B. Stancu I.-C. Buist A. Bird M. Wang P. Vanoosthuyse A.. (2016). Heterotypic seeding of Tau fibrillization by pre-aggregated Aβ provides potent seeds for prion-like seeding and propagation of Tau-pathology *in vivo*. Acta Neuropathol. 131, 549–569. 10.1007/s00401-015-1525-x 26739002 PMC4789256

[B128] Venegas C. Kumar S. Franklin B. S. Dierkes T. Brinkschulte R. Tejera D.. (2017). Microglia-derived ASC specks cross-seed amyloid-β in Alzheimer’s disease. Nature 552, 355–361. 10.1038/nature25158 29293211

[B129] Walker L. C. Callahan M. J. Bian F. Durham R. A. Roher A. E. Lipinski W. J. (2002). Exogenous induction of cerebral β-amyloidosis in βAPP-transgenic mice. Peptides 23, 1241–1247. 10.1016/s0196-9781(02)00059-1 12128081

[B130] Walsh D. M. Klyubin I. Fadeeva J. V. Cullen W. K. Anwyl R. Wolfe M. S.. (2002). Naturally secreted oligomers of amyloid β protein potently inhibit hippocampal long-term potentiation *in vivo*. Nature 416, 535–539. 10.1038/416535a 11932745

[B131] Walsh D. M. Lomakin A. Benedek G. B. Condron M. M. Teplow D. B. (1997). Amyloid β-protein fibrillogenesis. Detection of a protofibrillar intermediate. J. Biol. Chem. 272, 22364–22372. 10.1074/jbc.272.35.22364 9268388

[B132] Wang H.-W. Pasternak J. F. Kuo H. Ristic H. Lambert M. P. Chromy B.. (2002). Soluble oligomers of β amyloid (1–42) inhibit long-term potentiation but not long-term depression in rat dentate gyrus. Brain Res. 924, 133–140. 10.1016/s0006-8993(01)03058-x 11750898

[B133] Watts J. C. Condello C. Stöhr J. Oehler A. Lee J. DeArmond S. J.. (2014). Serial propagation of distinct strains of Aβ prions from Alzheimer’s disease patients. Proc. Natl. Acad. Sci. U S A 111, 10323–10328. 10.1073/pnas.1408900111 24982139 PMC4104857

[B134] Watts J. C. Giles K. Grillo S. K. Lemus A. DeArmond S. J. Prusiner S. B. (2011). Bioluminescence imaging of Aβ deposition in bigenic mouse models of Alzheimer’s disease. Proc. Natl. Acad. Sci. U S A 108, 2528–2533. 10.1073/pnas.1019034108 21262831 PMC3038719

[B135] Weller R. O. Massey A. Newman T. A. Hutchings M. Kuo Y. M. Roher A. E. (1998). Cerebral amyloid angiopathy: amyloid β accumulates in putative interstitial fluid drainage pathways in Alzheimer’s disease. Am. J. Pathol. 153, 725–733. 10.1016/s0002-9440(10)65616-7 9736023 PMC1853019

[B136] Xue W.-F. Hellewell A. L. Gosal W. S. Homans S. W. Hewitt E. W. Radford S. E. (2009). Fibril fragmentation enhances amyloid cytotoxicity. J. Biol. Chem. 284, 34272–34282. 10.1074/jbc.m109.049809 19808677 PMC2797196

[B137] Yang F. Uéda K. Chen P. Ashe K. H. Cole G. M. (2000). Plaque-associated α-synuclein (NACP) pathology in aged transgenic mice expressing amyloid precursor protein. Brain Res. 853, 381–383. 10.1016/s0006-8993(99)02207-6 10640638

[B138] Ye L. Fritschi S. K. Schelle J. Obermüller U. Degenhardt K. Kaeser S. A.. (2015a). Persistence of Aβ seeds in APP null mouse brain. Nat. Neurosci. 18, 1559–1561. 10.1038/nn.4117 26352792

[B139] Ye L. Hamaguchi T. Fritschi S. K. Eisele Y. S. Obermüller U. Jucker M.. (2015b). Progression of seed-induced Aβ deposition within the limbic connectome. Brain Pathol. 25, 743–752. 10.1111/bpa.12252 25677332 PMC4530099

[B140] Ye L. Rasmussen J. Kaeser S. A. Marzesco A.-M. Obermüller U. Mahler J.. (2017). Aβ seeding potency peaks in the early stages of cerebral β-amyloidosis. EMBO Rep. 18, 1536–1544. 10.15252/embr.201744067 28701326 PMC5579388

[B141] Ziegler-Waldkirch S. d’Errico P. Sauer J.-F. Erny D. Savanthrapadian S. Loreth D.. (2018). Seed-induced Aβ deposition is modulated by microglia under environmental enrichment in a mouse model of Alzheimer’s disease. EMBO J. 37, 167–182. 10.15252/embj.201797021 29229786 PMC5770788

